# Artistic occupations are associated with a reduced risk of Parkinson’s disease

**DOI:** 10.1007/s00415-015-7828-y

**Published:** 2015-07-03

**Authors:** Charlotte A. Haaxma, George F. Borm, Dimitri van der Linden, Arnoud C. Kappelle, Bastiaan R. Bloem

**Affiliations:** Department of Neurology, Donders Institute for Brain, Cognition and Behaviour, Radboud University Medical Centre, PO Box 9101, 6500 HB Nijmegen, The Netherlands; Parkinson Centre Nijmegen, Radboud University Medical Centre, Nijmegen, The Netherlands; Department of Epidemiology, Biostatistics and HTA, Radboud University Nijmegen Medical Centre, Nijmegen, The Netherlands; Institute of Psychology, Erasmus University Rotterdam, Rotterdam, The Netherlands

**Keywords:** Parkinson’s disease, Case–control study, Premotor, Occupations, Artistic

## Abstract

Parkinson’s disease (PD) is preceded by a premotor phase of unknown duration. Dopaminergic degeneration during this phase may lead to subtle cognitive and behavioural changes, such as decreased novelty seeking. Consequently, premotor subjects might be most comfortable in jobs that do not require optimal dopamine levels, leading to an overrepresentation in structured and predictable occupations, or an underrepresentation in artistic occupations. In a case–control study, 750 men with PD (onset ≥40 years) and 1300 healthy men completed a validated questionnaire about their lifetime occupational status. Occupations were classified using the RIASEC model. Odds ratios (ORs) were calculated for the conventional and artistic categories, both for the most recent occupation before symptom onset, and for the very first occupation. Because farming has been associated with a PD risk, ORs were calculated separately for farming. A reduced risk of PD was found for men with an artistic occupation late in life (OR 0.14, 95 % CI 0.04–0.53), while an artistic first occupation did not prevent PD (OR 0.72, CI 0.32–1.59). Conventional occupations showed no increased risk (recent: OR 1.07, CI 0.70–1.64; first: OR 1.14, CI 0.77–1.71). In support of previous reports, farming was associated with an increased risk of PD (recent: OR 2.6, CI 1.4–4.6; first: OR 2.7, CI 1.6–4.5). PD patients were older than controls, but various statistical corrections for age all lead to similar results. Artistic occupations late in life are associated with a reduced risk of subsequent PD, perhaps because this reflects a better preserved dopaminergic state. No initial occupation predicted PD, suggesting that the premotor phase starts later in life.

## Introduction

Parkinson’s disease (PD) is preceded by a premotor phase of unknown duration and onset, in which symptoms such as REM sleep behaviour disorder, hyposmia and depression may become present [1, 2]. However, the hypodopaminergic state that is at least partly related to these symptoms does not cause motor signs yet. Here, we use a new approach to probe the premotor phase and to examine its possible presence in early adulthood. We hypothesize that a mild hypodopaminergic state in premotor subjects induces subtle cognitive and behavioural changes (e.g. decreased novelty seeking and creativity), which may influence their occupational preferences and abilities prior to the diagnosis of PD. Several occupations have been linked with an increased risk of PD [3, 4]. Some of these, such as farming and welding, have been studied in the context of exposure to environmental toxins [5]. However, their exact relationship with PD is still unclear, and this is even more true for a variety of other occupations, such as teaching or ICT (information and communication technology) work. Perhaps, occupations with an increased risk of PD reflect a preference for jobs that do not require optimal dopaminergic function.

Low dopamine levels and low D_2_-receptor binding have been associated with a lack of novelty seeking in PD patients [6]. In turn, low novelty seeking scores in PD correlated with a reflective, rigid, loyal, frugal and orderly personality [7]. Following dopaminergic treatment, however, de novo artistic skills emerged [8]. From this perspective, premotor subjects may be overrepresented in structured, predictable occupations or underrepresented in artistic occupations. To investigate this, we used the RIASEC model [9], a validated method to classify occupations into six categories linked to personality characteristics (Table 1): realistic (R), investigative (I), artistic (A), social (S), enterprising (E), and conventional (C). Artistic jobs have been related to the personality trait ‘openness to new experience’ [10], which in turn is associated with high levels of central dopamine [11]. We focused on the two most contrasting RIASEC categories, i.e. ‘artistic’—characterized by creativeness, expressiveness and a disorderly work pattern—versus ‘conventional’—requiring high levels of structure, routines, and a clear direction.

**Table 1:** The RIASEC model [9] summarized

R: realistic	Auto mechanic, aircraft controller, surveyor, electrician, farmer, etc Have mechanical and athletic abilities Like to work outdoors and with tools and machines Generally like working with things more than with people Honest, humble, natural, persistent, practical, shy, and thrifty
I: investigative	Biologist, chemist, geologist, laboratory assistant, medical technician, etc Have math and science abilities Like to work alone and to solve problems Like to explore and understand things, rather than persuade others or sell things Curious, independent, modest, precise, rational, reserved, and smart
A: artistic	Musician, stage director, dancer, decorator, actor, writer, etc Have artistic skills, enjoy creating original work, have good imagination Like working with creative ideas and self-expression more than routines and rules Disorderly, expressive, idealistic, impractical, independent, open, and original
S: social	Teacher, speech therapist, religious worker, counselor, nurse etc Like to be around other people, are interested in how people get along, like to help other people with their problems Like helping and teaching people more than doing mechanical or technical activities Convincing, friendly, generous, helpful, idealistic, kind, patient, tactful, warm
E: enterprising	Sports promoter, TV producer, business executive, salesperson, manager, etc Have leadership and public speaking abilities, are interested in money and politics, like to influence people Like persuading or directing others, more than working on scientific or complicated topics Agreeable, ambitious, dominant, energetic, outgoing, pleasure-seeking, popular, and self-confident
C: conventional	Bookkeeper, financial analyst, banker, tax expert, secretary, etc Have clerical and math abilities Like to work indoors and to organize things Like to follow orderly routines and meet clear standards, avoiding work that does not have clear directions Careful, efficient, orderly, persistent, practical, and thrifty

## Methods

### Participants

In a case–control design, 750 PD patients (UK Brain Bank Criteria) [12], with an onset of motor symptoms at age 40 or above, were recruited from the Radboud University Nijmegen Medical Centre as well as from 15 community hospitals in a wide area surrounding Nijmegen. The patients were selected upon review of medical charts, excluding subjects with cognitive decline (MMSE <27) or with physical or psychiatric illnesses that could have influenced their job opportunities. 1300 healthy controls were derived from the Nijmegen Biomedical Study, a large population-based cross-sectional survey in and around Nijmegen [13]. We included men only, to exclude sex-related differences in job opportunities in the elderly population. All participants gave informed consent for data sharing. The study design was approved by the local ethics committee.

### Data acquisition

Patients and controls completed a structured questionnaire about their lifetime occupational status, years and level of education, and comorbidity. Patients also reported the subjective onset of their motor symptoms as well as their reasons to change or end a job. A research assistant who was blinded to the study aims classified each occupation according to the RIASEC system. This produces a three-letter code for every single occupation, related to the main personality characteristics associated with the occupation, e.g. ERS, RIA, and so on. In this study, we focused on the first letter of this code as it represents the occupation’s most dominant feature.

### Statistical methods

One hundred seventy-four questionnaires (PD: *n* = 57/750; controls: *n* = 117/1300) were not completed adequately and therefore excluded from the analyses. There were no major differences between both databases (PD and controls) in terms of basic characteristics except for a higher median age at the moment of completing the questionnaire in the PD group (69.4 vs. 51.2 years; Table 2). Therefore, we not only adjusted for age in all following calculations, but also performed additional analyses to identify any age effects (see end of this section).

**Table 2:** Subject characteristics and occupational distribution

	PD (*n* = 693)		Controls (*n* = 1183)	
Age (y) at time of questionnaire	69.4 ± 9.3 (42.0–90.0)		51.2 ± 7.4 (40.0–83.0)	
RIASEC categories; no. of occupations (%)	First occ.	Last occ.	First occ.	Last occ.
Realistic (R)	316 (51 %)	191 (34 %)	337 (31 %)	275 (23 %)
Investigative (I)	27 (4 %)	37 (7 %)	113 (16 %)	122 (10 %)
Artistic (A)	16 (3 %)	5 (1 %)	40 (4 %)	34 (3 %)
Social (S)	60 (10 %)	63 (11 %)	214 (19 %)	217 (18 %)
Enterprising (E)	102 (17 %)	171 (31 %)	222 (20 %)	355 (30 %)
Conventional (C)	97 (16 %)	91 (16 %)	176 (16 %)	180 (15 %)

We had two primary hypotheses: (1) a low PD risk for artistic occupations and (2) a high PD risk for conventional occupations. Logistic regression analyses were performed on the very first occupation and on the most recent occupation, which, in case of the patients, was the last occupation prior to the self-reported symptom onset. The primary analysis included the factors ‘artistic occupation (A; yes/no)’, ‘conventional occupation (C; yes/no)’ and ‘age’. Because we evaluated two hypotheses in the primary analysis, we used a Bonferroni correction and considered *p* < 0.025 statistically significant. In a secondary analysis, we investigated the role of educational level by including this as an additional factor in the logistic regression and by calculating the interaction between education and occupation. This led to *p* > 0.20 for the interaction term. We carried out separate analyses for subjects with educational levels up to intermediate vocational training, and those above.

We estimated odds ratios (ORs) for the artistic (A) occupations versus each of the remaining five occupational categories (R, I, S, E, C) with respect to their association with PD.

Since previous reports identified farming as a risk factor for PD, the OR of farming versus all other occupations was calculated as a validation of our data set in relationship with those previous reports. We repeated the primary analysis, both with farming as additional factor and without farming. We did not aim to prove or investigate the relationship between PD and farming in the light of toxin exposure.

We repeated the analyses restricted to subjects from the Nijmegen area to verify whether this specific geographical area would yield different results from the group in total.

With respect to the substantial age difference between PD and controls, we carried out the following additional calculations. We challenged the main assumption of linearity on the log odds scale in the logistic regression. When allowing for non-linearity, however, the effects remained almost unchanged. In particular, we performed an analysis with age, [age]^2^ and [age]^3^ as covariables, thus allowing for an effect that could change direction even up to two times. We also ran the analysis stratifying for age classes of 5 years. These ORs were then pooled and presented in an overall OR. Such an analysis does not assume any particular shape of the relationship between age, risk of PD or occupation. The mean age difference between PD and controls within the age classes was 0.02 years. We chose not to make the samples age-matched, as such post hoc matching would be rather arbitrary and would have led to a data set far too small for any reliable conclusion. Finally, we explored whether the number of job changes correlated with the risk of PD. We hypothesized that more frequent changes could be associated with a lower PD risk.

## Results

**Fig. 1:**
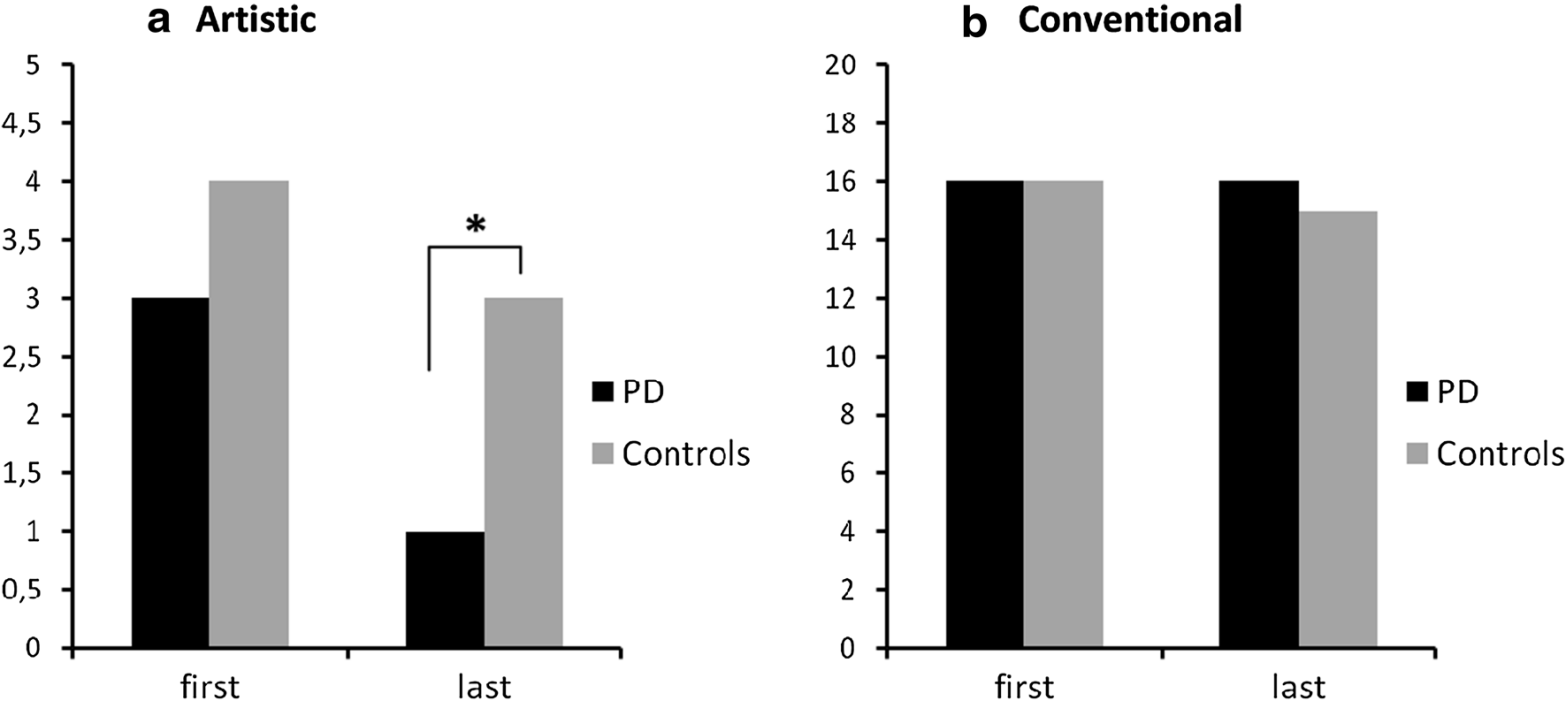
Percentage of artistic (**a**) and conventional (**b**) occupations within all first as well as all last (=most recent) occupations. PD patients are less likely than controls to have had an artistic occupation as their last occupation (*p = 0.004). For the conventional occupations, no substantial difference between PD and healthy subjects could be demonstrated

The distribution of the subjects across all occupations showed that artists represented a minority in both populations (between 1 and 4 %, Table 2). There was a reduced risk of subsequent PD for men with an artistic occupation late in life, compared to all other job categories (OR 0.14, CI 0.04–0.53, *p* = 0.004, Fig. 1). Even with this relatively small number of artistic occupations, the *p* value was robust enough to exclude that the effect resulted from pure chance.

In contrast to our hypothesis, conventional occupations late in life showed no increased risk of subsequent PD (OR 1.07, CI 0.70–1.64, *p* = 0.76, Fig. 1). Table 3 displays the ORs for artistic occupations in comparison to the other five RIASEC categories separately, which were all quite similar (OR 0.11–0.19). None of the first occupations showed an increased or decreased risk of PD, especially not the artistic (OR 0.72, CI 0.32–1.59, *p* = 0.41) and conventional categories (OR 1.14, CI 0.77–1.71, *p* = 0.51).

**Table 3:** Risk of PD, displayed for artistic jobs (A) as compared to all other occupational groups (R, I, S, E, C). Odds ratio’s (ORs) and 95 % confidence intervals (CI) after adjustment for age

Occupational category	OR (95 % CI)	
	First occupation	Last occupation
Realistic (R)		
Including farming	0.34 (0.21–0.56)	0.11 (0.03–0.43)
Excluding farming	0.38 (0.23–0.64)	0.13 (0.03–0.52)
Investigative (I)	1.00 (0.49–2.03)	0.19 (0.05–0.78)
Social (S)	0.66 (0.36–1.20)	0.18 (0.05–0.71)
Enterprising (E)	0.59 (0.34–1.02)	0.18 (0.04–0.56)
Conventional (C)	0.46 (0.26–0.81)	0.13 (0.03–0.52)
Age difference at questionnaire	0.80 (0.78–0.82)	

In support of previous research [15], an increased risk of PD was found for farming, which belongs to the R category (last: OR 2.6, CI 1.4–4.6; first: OR 2.7, CI 1.6–4.5). When repeated without farming, the ORs changed less than 0.02. Excluding other occupations in which toxin exposure could be present changed the ORs by less than 0.03.

Adding educational level as a covariable changed the ORs by less than 0.04. There was a non-significant interaction between education and occupation. The primary ORs diminished by half for the lower education group and doubled for those with higher education but all remained below 0.5 and, most importantly, the confidence intervals were very wide.

The analyses with age, [age]^2^ and [age]^3^ as covariables, as well as the analysis stratifying for age classes of 5 years, produced results closely matching the primary findings. The number of job changes did not correlate significantly with the risk of PD.

## Discussion

The present study shows a reduced risk of future PD for men with an artistic job late in life. One possible interpretation of this finding could be that artistic abilities are associated with a better preserved dopaminergic state, which postpones or is incompatible with the development of parkinsonian signs. This would be in line with previous reports of a positive relationship between artistic productivity and dopaminergic activity, especially after initiation of dopaminergic drug therapy [16–18]. Artistic creativity can also be enhanced by drugs such as cocaine or amphetamines, which stimulate the mesolimbic dopaminergic system, affecting reward responses and addiction behaviour [19, 20]. This may explain why artists have an increased risk of developing the dopamine dysregulation syndrome [16]. Conversely, artistic skills can be turned off by left subthalamic nucleus stimulation [21], and exploratory behaviour decreases in case of dopamine-depleting lesions in the nucleus accumbens or ventral tegmentum [22]. In parallel, our findings may support the hypothesis that a gradually declining dopamine level during the premotor phase of PD influences a subject’s occupational preference or abilities, and consequently reduces the likelihood of having an artistic occupation in the years before diagnosis.

Contrary to the second part of our hypothesis, conventional jobs were not associated with an increased risk of PD. This may be explained by the fact that the RIASEC classification—although a validated approach to categorize occupations as linked to personality characteristics [9]—is not an established approach to probe the dopaminergic state of subjects. Indeed, behaviour is determined not only by dopamine, but also by, e.g. noradrenalin, which is associated with harm avoidance [14]. However, the observed effect of the artistic occupations remained present after correction for potential confounders. Moreover, the RIASEC approach confirmed the previously established link between farming and PD, with a comparable risk (OR 2.6) as found by others (OR 2.5) [15]. This does not prove any causality but may allow us to compare the results of this research with previous reports.

No initial occupation predicted subsequent PD, suggesting that the premotor phase starts later in life. Despite the large number of subjects in our study, the low frequency of artistic jobs prevented us from determining at which point in time these conveyed to a lower risk of developing PD.

A few more remarks should be made with respect to this study’s limitations. First, we should point out that the age difference between cases and controls was substantial. We used various statistical adjustment methods to take this into account, and all types of analyses lead to similar results. However, there is understandably no guarantee that this post hoc statistical correction will lead to completely unbiased results. We can therefore not fully exclude the possibility that the results that we have found may have been partially incorrect; consequently, the results of our study must only be seen as indicative. A replication of the study is definitely necessary to draw more definite conclusions. Second, the reasons for people to change or end a job were diverse and difficult to interpret, mainly because of bias issues (e.g. recall bias and the possibility that patients themselves may not be able to correctly identify and appreciate the various reasons that could have led to changing or ending a job). Therefore, we chose not to run statistics on these data. We did not investigate the effect of socioeconomic status, which may have influenced (the freedom of) career choices differently for higher versus lower classes. However, socioeconomic status is closely related to educational level and occupation, which we did investigate extensively. Finally, this study focused on men only while important gender differences have been found in previous PD research. As a result, conclusions from the present study cannot be extrapolated to women. These issues should be a topic of future research.

## References

[1] Chaudhuri KR, Odin P (2010). The challenge of non-motor symptoms in Parkinson’s disease. Review. Prog Brain Res.

[2] Martinez-Martin P (2011). The importance of non-motor disturbances to quality of life in Parkinson’s disease. J Neurol Sci.

[3] Goldman SM, Tanner CM, Olanow CW (2005). Occupation and parkinsonism in three movement disorders clinics. Neurology.

[4] Park J, Yoo CI, Sim CS (2005). Occupations and Parkinson’s disease: a multi-center case–control study in South Korea. Neurotoxicology.

[5] Elbaz A, Moisan F (2008). Update in the epidemiology of Parkinson’s disease. Curr Opin Neurol.

[6] Kaasinen V, Aalto S, Nagren K (2004). Insular dopamine D(2) receptors and novelty seeking personality in Parkinson’s disease. Mov Disord.

[7] Menza MA, Golbe LI, Cody RA (1993). Dopamine-related personality traits in Parkinson’s disease. Neurology.

[8] Kulisevsky J, Pagonabarraga J, Martinez-Corral M (2009). Changes in artistic style and behaviour in Parkinson’s disease: dopamine and creativity. J Neurol.

[9] Holland JL (1973) Making vocational choices: a theory of careers. Prentice-Hall, Englewood Cliffs, New Jersey. [Revised version: Holland, JK (1997) Making vocational choices: a theory of vocational personalities and work environments. 3. Odessa, Fl: PAR]

[10] Schinka JA, Dye DA, Curtiss G (1997). Correspondence between five-factor and RIASEC models of personality. J Pers Assess.

[11] DeYoung CG, Peterson JB, Higgins DM (2005). Sources of openness/intellect: cognitive and neuropsychological correlates of the fifth factor of personality. J Pers.

[12] Hughes AJ, Daniel SE, Kilford L (1992). Accuracy of clinical diagnosis of idiopathic Parkinson’s disease: a clinico-pathological study of 100 cases. J Neurol Neurosurg Psychiatry.

[13] Hoogendoorn EH, Hermus AR, de Vegt F (2006). Thyroid function and prevalence of anti-thyroperoxidase antibodies in a population with borderline sufficient iodine intake: influences of age and sex. Clin Chem.

[14] Menza MA, Forman NE, Goldstein HS (1990). Parkinson’s disease, personality, and dopamine. J Neuropsychiatry Clin Neurosci.

[15] Rugbjerg K, Harris MA, Shen H (2011). Pesticide exposure and risk of Parkinson’s disease—a population-based case–control study evaluating the potential for recall bias. Scand J Work Environ Health.

[16] Schwingenschuh P, Katschnig P, Saurugg R (2010). Artistic profession: a potential risk factor for dopamine dysregulation syndrome in Parkinson’s disease?. Mov Disord.

[17] Kulisevsky J, Pagonabarraga J, Martinez-Corral M (2009). Changes in artistic style and behaviour in Parkinson’s disease: dopamine and creativity. J Neurol.

[18] Walker RH, Warwick R, Cercy SP (2006). Augmentation of artistic productivity in Parkinson’s disease. Mov Disord.

[19] Bressan RA, Crippa JA (2005). The role of dopamine in reward and pleasure behaviour—review of data from preclinical research. Acta Psychiatr Scand Suppl.

[20] Wise RA (2002). Brain reward circuitry: insights from unsensed incentives. Neuron.

[21] Drago V, Foster PS, Okun MS (2009). Turning off artistic ability: the influence of left DBS in art production. J Neurol Sci.

[22] Stellar JR, Stellar E (1985). The neurobiology of motivation and reward.

